# Serum concentrations of cortisol, interleukin 6, leptin and adiponectin predict stress induced insulin resistance in acute inflammatory reactions

**DOI:** 10.1186/cc7152

**Published:** 2008-12-17

**Authors:** Michael Lehrke, Uli C Broedl, Ingeborg M Biller-Friedmann, Michael Vogeser, Volkmar Henschel, Kirsten Nassau, Burkhard Göke, Erich Kilger, Klaus G Parhofer

**Affiliations:** 1 Department of Internal Medicine II, University of Munich, Grosshadern Campus, Marchioninistr 15, 81377 Munich, Germany; 2 Department of Clinical Chemistry, University of Munich, Grosshadern Campus, Marchioninistr 15, 81377 Munich, Germany; 3 Department of Medical Informatics, University of Munich, Grosshadern Campus, Marchioninistr 15, 81377 Munich, Germany; 4 Department of Anesthesia; University of Munich, Grosshadern Campus, Marchioninistr 15, 81377 Munich, Germany

## Abstract

**Introduction:**

Inflammatory stimuli are causative for insulin resistance in obesity as well as in acute inflammatory reactions. Ongoing research has identified a variety of secreted proteins that are released from immune cells and adipocytes as mediators of insulin resistance; however, knowledge about their relevance for acute inflammatory insulin resistance remains limited. In this study we aimed for a clarification of the relevance of different insulin resistance mediating factors in an acute inflammatory situation.

**Methods:**

Insulin resistance was measured in a cohort of 37 non-diabetic patients undergoing cardiac surgery by assessment of insulin requirement to maintain euglycaemia and repeated measurements of an insulin glycaemic index. The kinetics of cortisol, interleukin 6 (IL6), tumour necrosis factor α (TNFα), resistin, leptin and adiponectin were assessed by repeated measurements in a period of 48 h.

**Results:**

Insulin resistance increased during the observation period and peaked 22 h after the beginning of the operation. IL6 and TNFα displayed an early increase with peak concentrations at the 4-h time point. Serum levels of cortisol, resistin and leptin increased more slowly and peaked at the 22-h time point, while adiponectin declined, reaching a base at the 22-h time point. Model assessment identified cortisol as the best predictor of insulin resistance, followed by IL6, leptin and adiponectin. No additional information was gained by modelling for TNFα, resistin, catecholamine infusion rate, sex, age, body mass index (BMI), operation time or medication.

**Conclusions:**

Serum cortisol levels are the best predictor for inflammatory insulin resistance followed by IL6, leptin and adiponectin. TNFα, and resistin have minor relevance as predictors of stress dependent insulin resistance.

## Introduction

The Western lifestyle has created a pandemic of obesity, which has dramatically increased the prevalence of insulin resistance and diabetes mellitus. Efforts to understand the linkage between the accumulation of body fat and the occurrence of insulin resistance have identified a variety of adipose tissue derived secreted proteins as mediators for insulin resistance. Some of these so-called adipokines such as leptin or adiponectin are adipocyte specific, while other mainly inflammatory cytokines are secreted by immune cells that infiltrate the adipose tissue in an obesity dependent manner. Diabetes has therefore also been considered a chronic inflammatory disease [1]. The relevance of inflammatory proteins as mediators of insulin resistance is not restricted to the chronic metabolic environment of obesity but also found in acute inflammatory reactions such as sepsis, which are marked by severe insulin resistance and often hyperglycaemia. Inflammatory cytokines such as tumour necrosis factor (TNF)α and interleukin (IL)6 activate signalling cascades including nuclear factor κB (NF-κB) and C-Jun N-terminal kinases (JNK), which inhibit insulin signalling by serine phosphorylation of insulin receptor substrate 1 (IRS-1) and thereby reduce translocation of the glucose transporter GLUT4 to the cell membrane [1]. Adipocyte specific regulators of insulin sensitivity include leptin, which primarily serves as a fuel storage sensor relevant for appetite regulation and thermogenesis [2]. Adiponectin, which promotes insulin sensitivity by activation of adenosine monophosphate-activated protein kinase (AMPK) and resistin, which has been identified as an adipocyte specific promoter of insulin resistance in mice [3].

While these factors were characterised in the chronic metabolic environment of obesity, our knowledge about their relevance as mediators of insulin resistance in acute inflammatory situations remains limited. Stress induced insulin resistance has classically been ascribed to increased serum levels of cortisol which promotes gluconeogenesis and inhibits peripheral glucose disposal in a stress dependent manner. However, TNFα, IL6, resistin or leptin are induced and adiponectin levels are reduced by inflammatory stimuli, which makes it likely that similar mechanisms are relevant in chronic and acute inflammation [4,5]. The comparable pattern of regulative proteins in the chronic environment of obesity and in acute inflammation suggests similar causative mechanisms of insulin resistance. Identification and characterisation of the most important pathways of insulin resistance remains crucial for the development of new therapeutic strategies. The relevance of tight glycaemic control is thereby not restricted to the treatment of diabetes but also crucial in acute inflammatory situations, where maintenance of euglycaemia improved perioperative outcome and reduced mortality in critically ill patients [6,7]. While obesity is a relative static cause of insulin resistance, characterised by low grade inflammation, we here decided to study the time course of insulin resistance following the acute intervention of cardiac surgery with extracorporeal circulation, which is a known inflammatory stimulus [8,9]. The aim of the current study was to classify the relevance of different insulin resistance mediating factors in direct comparison to each other.

## Materials and methods

We prospectively enrolled 37 non-diabetic patients scheduled for cardiac surgery with cardiopulmonary bypass and requirement of extracorporeal circulation. Patients were excluded from the study if they met the following criteria: pregnancy, diabetes mellitus, fasting glucose > 126 mg/dl, use of antidiabetic medication or glucocorticoids.

Patients were fasting since the evening of the preoperative day. Insulin resistance was recorded by the individual insulin requirements to maintain euglycaemia. Blood glucose was assessed on an hourly bases and insulin infusion rate consequently adjusted to maintain glucose levels between 80 and 126 mg/dl. In addition, repeated measurements of C peptide as an indicator of endogenous insulin production were recorded as well as insulin serum levels, representing the circulating sum of endogenously produced and exogenous applied insulin. An insulin glycaemic index was calculated at each time point (insulin × glucose/22.5). Blood samples were drawn directly before surgery (baseline), at arrival in the intensive care unit (ICU) (4 to 6 h time point), 6 h post arrival in the ICU (10 to 12 h time point) and the morning of the first and second postoperative days. As some patients were discharged to a normal ward at the first postoperative day, blood was only collected from 26 patients on the second postoperative day. Blood samples were stored on ice and directly centrifuged for serum collection. No glucose containing solutions were given during the day of the procedure, while all patients were started on a continuous infusion of glucose 10% with a rate of 10 ml/h at the morning of the first postoperative day. Low rate exogenous applied glucose did therefore only affect the last blood sampling at the second postoperative day. No additional parenteral or enteral nutrition was supplied during the observation period. The applied catecholamine doses were recorded at the blood collection time point in mg/h. The study protocol was approved by the Ethics Committee of the Ludwig-Maximilians-University Munich, Germany. All patients gave informed written consent.

### Laboratory procedures

Blood samples were stored at -70°C until analysis. Serum levels of TNFα, IL6, leptin, adiponectin and resistin were determined with a commercial enzyme-linked immunosorbent assay (R&D, Wiesbaden, Germany). Serum concentrations of cortisol, insulin, and C peptide were quantified using a multichannel immunoanalyzer based on electrochemiluminescence as the principle of signal generation (Roche Cobas, Elecsys 2010; Roche Diagnostics Mannheim, Germany) by the Department of Clinical Chemistry (Campus Grosshadern, University of Munich, Germany).

### Statistical analysis

Spearman correlation was performed for associations between baseline characteristics. A linear mixed effects model was fit to model the influence of the metabolic factors on insulin resistance (log insulin glycaemic index). The factors were included forward by a likelihood ratio test. A random effect per patient accounts for the subjective level of each patient (which includes the baseline levels) and for the dependence of the measurements within each patient. All measurements of a patient were used together with the time after surgery when they occurred (rounded to the nearest hour) Curves were fitted by the non-parametric locally-weighted scatterplot smoothing (LOWESS) smoother, which uses locally-weighted polynomial regression. Statistical analysis was performed using R 2.6.1.

## Results

Baseline characteristics of the study participants are shown in Table 1. All patients underwent open cardiac surgery with cardiopulmonary bypass.

**Table 1:** Baseline characteristics of the study population.

**Characteristics**	**n = 37**
Age (years)	69 (60 to 74)
Sex:	
Male	23
Female	14
Body mass index (kg/m^2^)	27.1 (23.1 to 29.9)
Hypertension	72%
Smoker (yes)	31%
Baseline laboratory profile:	
Glucose (mg/dl)	99 (91 to 107)
Insulin (uU/ml)	4.25 (1.9 to 6.7)
insulin glycaemic index	1.03 (0.45 to 1.77)
C Peptide (ng/ml)	1.6 (1.2 to 2.3)
Cortisol (ug/dl)	12.3 (7.4 to 16.1)
IL6 (pg/ml)	2 (2.0 to 2.45)
TNFα (pg/ml)	8.07 (6.37 to 19.14)
Resistin (ng/ml)	10.44 (7.94 to 14.5)
Leptin (pg/ml)	4,752 (2,714 to 10,636)
Adiponectin (ng/ml)	4,227 (2,917 to 7,190)
Medical treatment:	
Statin	67%
Beta blocker	78%
ACE-I or ARB	47%
Diuretics	56%
Aspirin	100%
Intervention:	
Operation time (min)	300 (270 to 330)
Heart/lung bypass time (min)	89 (74 to 106)

Values are presented as median (interquartile range) or proportions. ACE-I, angiotensin converting enzyme inhibitor; ARB, angiotensin II receptor blocker; Il, interleukin; TNF, tumour necrosis factor.

### Kinetics of insulin resistance

**Figure 1:**
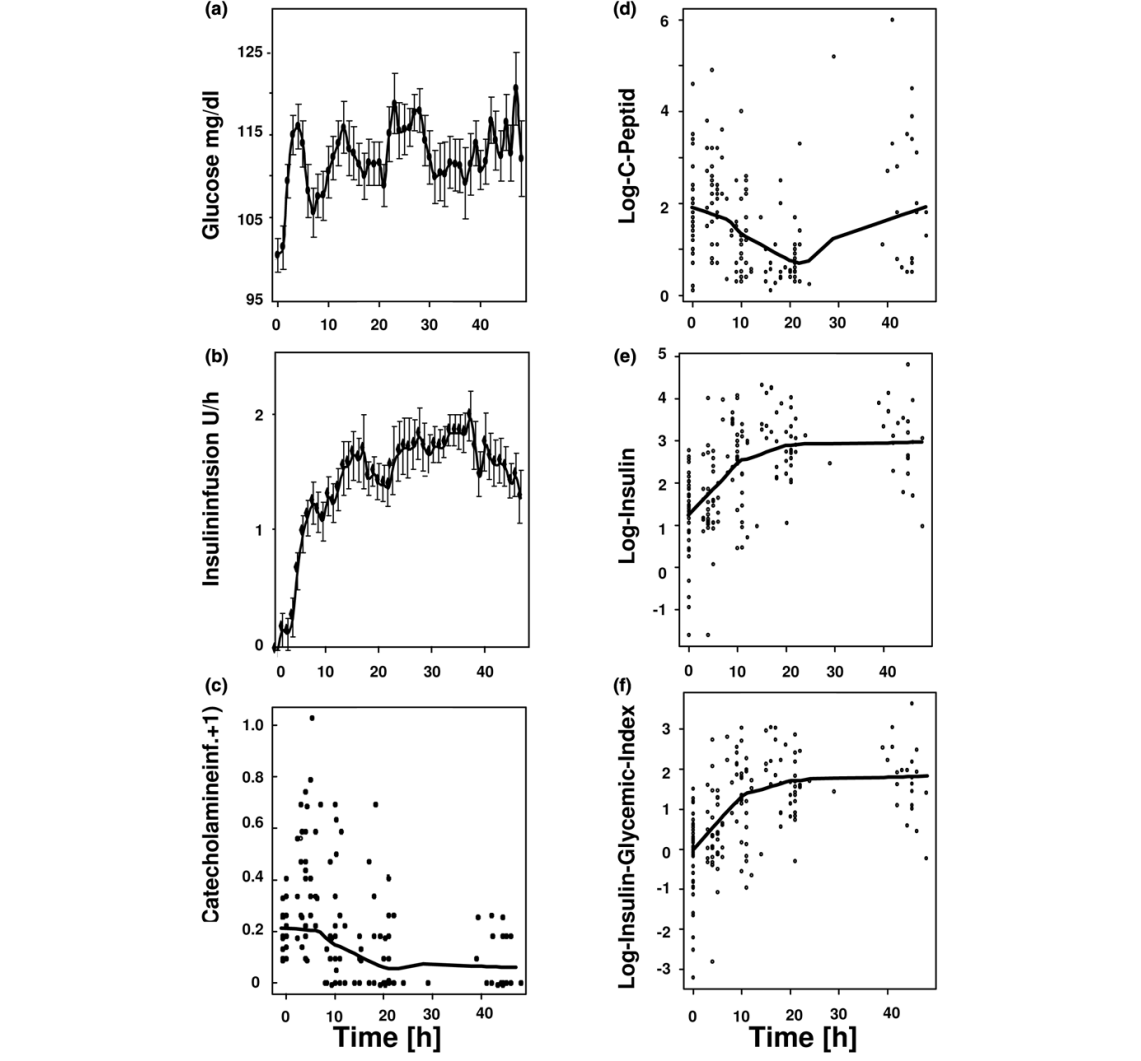
**Kinetics of insulin resistance**. Shown is the kinetic of serum glucose (a) and insulin infusion rate (b) during the observation period depicted as mean ± standard error of the mean (SEM). In addition, log (catecholamine infusion rate+1) (c), serum levels of log C peptide (d), and log insulin (e) log insulin glycaemic index (f) are depicted as a scatter plot with its locally-weighted scatterplot smoothing (LOWESS) estimation curve. The log scale was chosen for better presentation of outliers.

Blood glucose was monitored on an hourly basis throughout the observation period. All patients required insulin treatment to maintain euglycaemia. Consequently, blood glucose was kept stable throughout the observation period (Figure 1a), while insulin infusion rate increased with maximum concentrations required between the 17th and 38th hour of observation (Figure 1b), while required catecholamine doses declined throughout the observation period (Figure 1c). Blood samples were drawn at baseline, early postoperative, directly after submission to the ICU (marking a 4 to 6 h time point), 6 h after submission to the ICU (marking the 10 to 12 h time point) and the morning of the first and second postoperative days. C Peptide concentrations declined during the observation period, reaching a base at the 22 h time point, signifying a suppression of endogenous insulin production by exogenously applied insulin (Figure 1d). Serum insulin concentrations increased during the first 22 h of observation, following the course of exogenously applied insulin and remained stable thereafter (Figure 1e). To create a more specific parameter of insulin resistance that combines serum glucose with serum insulin levels, we decided to calculate an insulin glycaemic index (insulin × glucose/22.5) at each time point (Figure 1f). Consequently, the insulin glycaemic index increased during the first 22 h of the observation period and remained stable thereafter, again resembling the kinetics of exogenous applied insulin.

### Baseline characteristics of serum parameters

At baseline (preoperative blood sample) we found correlations between IL6, resistin and TNFα (IL6-resistin; r = 0.4; p < 0.01) (IL6-TNFα; r = 0.29; p < 0.05) (resistin-TNFα; r = 0.33; p < 0.05). In addition, baseline leptin and adiponectin were found to correlate positively or negatively with body mass index (BMI) (leptin-BMI; r = 0.5; p < 0.001) (adiponectin-BMI; r = -0.45; p < 0.01).

### Inflammatory kinetic of serum parameters

**Figure 2:**
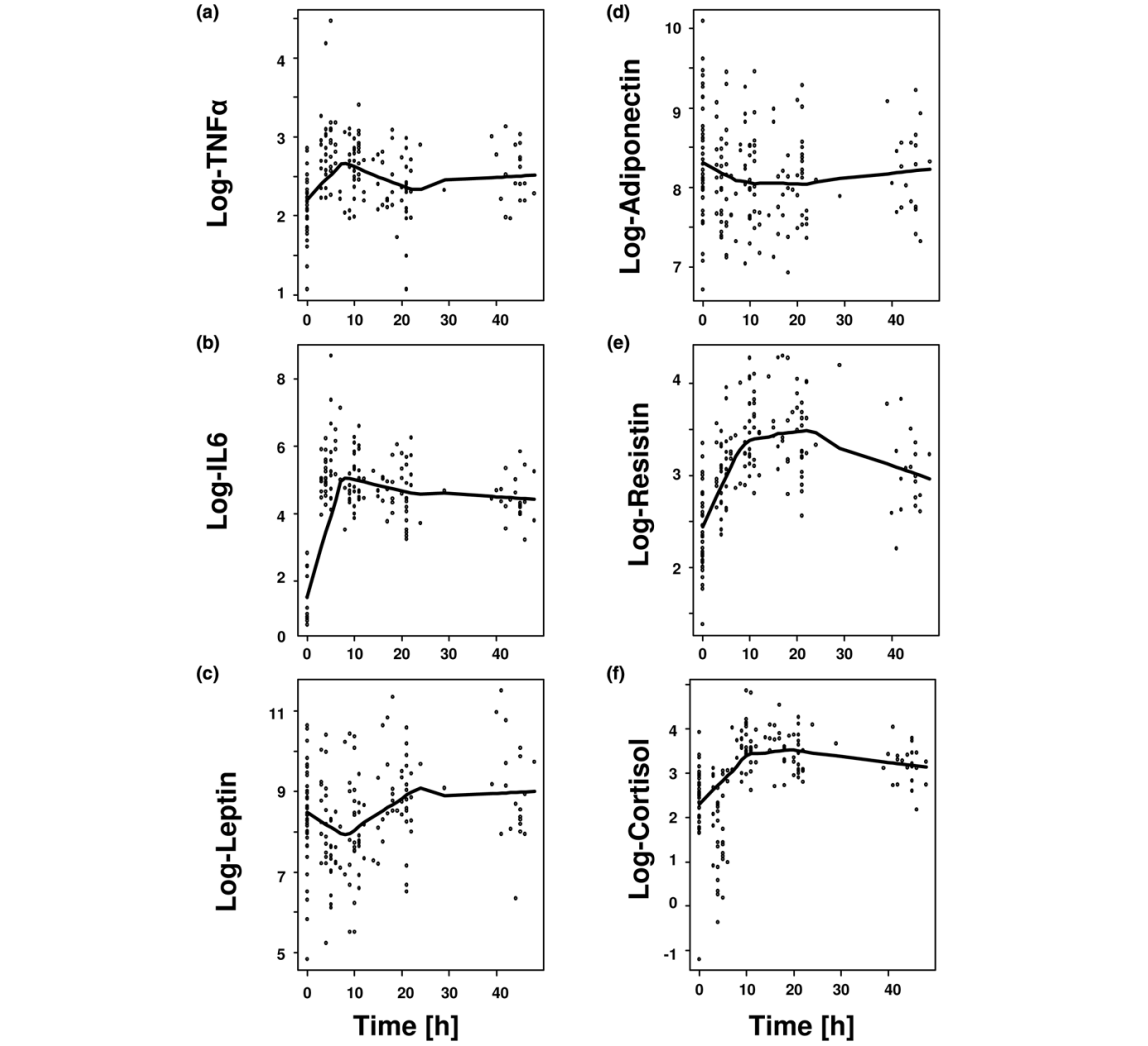
**Kinetics of serum parameters**. Shown is the kinetic of serum log tumour necrosis factor (TNF)α (a), log interleukin (IL)6 (b), log leptin (c), log adiponectin (d), log resistin (e) and log cortisol (f) during the observation period depicted as a scatter plot with its locally-weighted scatterplot smoothing (LOWESS) estimation curve. The log scale was chosen for better presentation of outliers.

During the observation period inflammatory cytokines rapidly increased with peak concentrations of TNFα and IL6 found at the 4 to 6 h time point (Figure 2a,b). Serum levels of leptin initially decreased, reaching a minimum at the 10 to 12 h time point to secondarily increase to supranormal levels peaking at the 20 to 22 h time point (Figure 2c). Adiponectin serum levels were repressed throughout the observation period reaching a minimum at the 20 to 22 h time point (Figure 2d). Resistin serum levels steadily increased to reach their maximum at the 20 to 22 h time point (Figure 2e). Cortisol serum levels increased during the observation period with maximum concentrations found at the 20 to 22 h time point (Figure 2f).

### Prediction of insulin resistance by serum parameters

We next asked which parameter would best predict insulin resistance. Using a linear mixed effects model we included the parameters by a forward selection using a likelihood ratio test to predict the individual insulin resistance as measured by the insulin glycaemic index. The model thereby includes the baseline and all following values of each parameter in a time dependent manner for each patient. The kinetic of each parameter was than analyzed for its relevance to predict insulin resistance in the same patient.

Serum cortisol was found to be the strongest predictor for the insulin glycaemic index (F = 104.26; p < 0.0001), followed by IL6 (F = 27.63; p < 0.0001), leptin (F = 18.12; p < 0.0001) and adiponectin (F = 4.7; p < 0.05) (Table 2). Additional modelling for TNFα, resistin, catecholamine infusion rate, age, gender, BMI, operation time, heart/lung bypass time or medication did not further improve the model, suggesting minor contribution of these parameters to the development of insulin resistance in our model.

**Table 2:** Prediction of insulin glycaemic index by different serum parameters using the equation: *E*(log(*Homa*_*ij*_)|*b*_*i*_) = *β*_0 _+ *β*_1 _log(*Corti*)_*ij *_+ *β*_2 _log(*Il*6)_*ij *_+ *β*_3 _log(*lep*)_*ij *_+ *β*_4 _log(*adi*)_*ij *_+ *b*_*i*_,   *i *= 1,..., 37, *j *= 1,..., 5 Where i indicates patients 1 to 37 and j indicates the different time points (1 to 5) assessed in each patient

	**β**	**Estimate, confidence interval**	**F value**	**p Value**
Intercept	β_0_	-1.25 (-4.10 to 1.60)	84.75	< 0.0001
Log cortisol	β_1_	0.57 (0.43 to 0.71)	104.26	< 0.0001
Log IL6	β_2_	0.20 (0.12 to 0.28)	27.63	< 0.0001
Log leptin	β_3_	0.29 (0.15 to 0.43)	18.12	< 0.0001
Log adiponectin	β_4_	-0.37 (-0.64 to -0.03)	4.7	0.03

The likelihood ratio test was used to model for insulin glycaemic index. IL, interleukin.

## Discussion

To better understand the relevance of different mediators of insulin resistance we have performed kinetic studies in an acute inflammatory setting in humans. Inflammation caused by cardiac surgery increased insulin resistance in a time dependent manner which was paralleled by an induction of cortisol, TNFα, IL6, resistin and leptin while adiponectin serum levels were decreased. Testing the relevance of each parameter to predict insulin resistance we found best performance for serum cortisol followed by serum IL6, leptin and adiponectin. No additional information was gained by modelling for TNFα, resistin, catecholamine infusion rate, gender, BMI, operation time, heart/lung bypass time or medication, suggesting a minor relevance of these parameters for inflammatory insulin resistance in our model.

Cortisol is the major adaptive signalling regulator of stress. Cortisol increases glucose availability by augmentation of hepatic glucose production via transcriptional and post-transcriptional activation of gluconeogenic enzymes including glucose-6-phosphatase and phosphoenolpyruvate [10]. In addition, cortisol inhibits glucose uptake and utilisation by peripheral tissues [11]. By contrast, cortisol excess impairs glucose tolerance and causes diabetes. Our model therefore confirms the dominating role of cortisol as a regulator of stress dependent insulin resistance.

An ongoing debate considers the relevance of IL6 as a mediator of insulin resistance in humans [12,13]. Initial evidence for a functional interplay was created by increased serum levels of IL6 in obesity in which IL6 was found to be associated with insulin resistance [14-17]. Interventional studies, using acute or chronic application of IL6, confirmed its potential to induce insulin resistance [18,19], while antibody-neutralisation experiments of IL6 were found to do the opposite [20]. Mechanistically, IL6 was found to impair insulin signalling primarily in the liver by induction of suppressor of cytokine signalling 3 (SOCS3) and inhibitory IRS-1 phophorylation [21]. However, application of IL6 to healthy volunteers recently failed to cause insulin resistance in humans [22] and was even associated with improved muscular glucose disposal and decreased endogenous glucose production, which was attributed to IL6 dependent activation of AMPK [21]. These studies were however limited by a confined observation period of a maximum 3 h, which was probably not sufficient to detect deleterious effects of IL6 on insulin sensitivity. Our study argues for a relevant role of IL6 as a mediator of stress dependent insulin resistance in the acute inflammatory setting in humans.

Leptin is a predominant regulator of energy metabolism with additional immune regulatory functions [23]. Leptin secretion is increased by inflammatory stimuli and promotes cellular and humoural immune responses. Leptin thereby stimulates the secretion of TNFα and IL6 from mononuclear cells and orchestrates in the cytokine network of inflammation [24]. Consistently, leptin deficiency impairs immune function, making the hosts more vulnerable to infectious disease [25,26]. Reports of leptin dependent effects on insulin sensitivity have been conflicting [27]. In a variety of studies, leptin administration was found to improve insulin sensitivity independently of body weight reducing effects [28,29]. However, other studies found no effect of leptin on glucose homeostasis [30], while additional studies reported leptin dependent inhibition of insulin secretion [31] and insulin signalling in isolated hepatocytes, myocytes or adipocytes [27]. Interestingly, leptin levels were suppressed at early time points in our model but secondarily increased to suprabasal levels at later time points and overall positively associated with insulin resistance. These results suggest a contributive effect of leptin to insulin resistance in inflammatory settings. Alternatively, our observations could also signify the occurrence of inflammation dependent leptin resistance, provoking a contra regulatory increase of leptin secretion [32]. Future studies are needed to clarify the relevance of inflammation dependent leptin secretion for insulin resistance.

Adiponectin has been identified as an insulin sensitising adipocyte derived protein, which is decreased in obesity [3]. Adiponectin deficient mice are prone to diet induced obesity and insulin resistance, which can be reversed by adiponectin treatment [33]. In humans low adiponectin was found to be closer associated with insulin resistance than adiposity [34]. Consistent with others we found inflammation dependent repression of adiponectin serum levels in our model which was modestly associated with insulin resistance, suggesting a contribution of adiponectin reduction to stress dependent insulin resistance [4]. The inflammatory regulation of adiponectin and leptin and their association to glucose metabolism in our model suggests a direct contribution of the adipose tissue to stress dependent glucose metabolism. Although, these adipocyte derived signals are presumably less relevant than cortisol as major metabolic adaptor to stress.

Resistin has been established as an adipocyte derived mediator of insulin resistance in mice [35]. Some but not all studies found a similar functional role in humans [3]. In contrast to mice, resistin is expressed by mononuclear cells in humans and stimulated in an inflammation dependent manner [36]. This prompted the hypothesis of resistin being a prominent mediator of inflammatory insulin resistance in humans, which we could not confirm in this study.

Although TNFα has been found to be a mediator of insulin resistance in acute and chronic models of inflammation [22,37], TNFα was not identified as an influential mediator in our model. This discrepancy could be explained by indirect effects of TNFα on insulin resistance, potentially requiring the induction of an additional TNFα dependent mediator of insulin resistance such as cortisol [38].

Better understanding of the relevant mediators of inflammatory insulin resistance will provide potentially clinical relevant information. Following the concept of relative adrenal insufficiency substitution of hydrocortisone has widely been used in critically ill patients. A recent study has now re-evaluated this approach and reported minor survival rates in hydrocortisone treated patients with septic shock due to infectious causes [39]. Taking the beneficial effects of normoglycaemia in the same patient collective, induction of cortisol dependent insulin resistance and hyperglycaemia might contribute to the observed detrimental effects [6,7].

This study has several limitations. We used an insulin glycaemic index to quantify insulin resistance, which is less precise than an insulinic clamp, the gold standard for the assessment of insulin resistance. However, the extended observation period limited the use of a clamp setting in our study. Future experiments are needed to confirm our results under clamp conditions.

The proposed model only offers associations but cannot provide causal relationship between the different parameters and insulin resistance. In addition, we cannot role out that other mediators of insulin resistance such as catecholamines, glucagon or growth hormone might also have contributive effects. The study is further limited by a relatively small sample size. Further studies in larger cohorts are needed to confirm the obtained results and further differentiate the relevance of specific factors preferentially under insulinic clamp conditions.

## Conclusion

Serum cortisol levels are the best predictor for inflammatory insulin resistance followed by IL6, leptin and adiponectin. TNFα, and resistin have minor relevance as predictors of stress dependent insulin resistance.

## Key messages

• Cortisol is the best predictor of stress induced insulin resistance during the course of cardiac surgery.

• Insulin resistance is further predicted by IL6, leptin and adiponectin.

• TNFα, and resistin do not predict stress induced insulin resistance in our model.

## Abbreviations

ACE-I: angiotensin converting enzyme inhibitor; ARB: angiotensin II receptor blocker; AMPK: adenosine monophosphate-activated protein kinase; GLUT4: glucose transporter 4; IL6: interleukin 6; IRS-1: insulin receptor substrate 1; JNK: C-Jun N-terminal kinase; NF-κB: nuclear factor κB; SOCS3: suppressor of cytokine signalling 3; TNFα: tumour necrosis factor α.

## Competing interests

The authors declare that they have no competing interests.

## Authors' contributions

ML contributed to study design, execution, and manuscript preparation. UCB contributed to study design, manuscript editing, IMBF sample collection and ELISA performance. MV contributed to insulin, C peptide and cortisol assays. VH contributed to statistical analysis. KN contributed to patient recruitment. BG contributed to study design and manuscript editing. EK contributed to patient recruitment. KP contributed to study design and manuscript editing.
